# Suicide attempts by poisoning recorded at a toxicological information and assistance center: a cross-sectional study, Fortaleza, 2022

**DOI:** 10.1590/S2237-96222025v34e20240885.en

**Published:** 2025-08-11

**Authors:** Hendyelle Rodrigues Ferreira e Silva, Wanderley Pinheiro de Holanda, Karla do Nascimento Magalhães, Dário Luis do Nascimento Magalhães, Alan Queiroz de Souza Santos, Maria Augusta Drago Ferreira

**Affiliations:** 1 Escola de Saúde Pública do Ceará, Residência Multiprofissional em Urgência e Emergência, Fortaleza, CE, Brazil; 2 Perícia Forense do Estado do Ceará, Núcleo de Toxicologia Forense, Fortaleza, CE, Brazil; 3 Instituto Doutor José Frota, Centro de Informação e Assistência Toxicológica, Fortaleza, CE, Brazil; 4 Universidade Estadual do Ceará, Centro de Ciências da Saúde, Fortaleza, CE, Brazil; 5 Universidade Federal do Ceará, Departamento de Farmácia, Fortaleza, CE, Brazil

**Keywords:** Suicidal ideation, Poisoning, Toxicology, Epidemiology, Cross-Sectional Analysis, Ideación Suicida, Intoxicación, Toxicología, Epidemiología, Análisis Transversal

## Abstract

**Objective:**

To outline the epidemiological profile of poisonings due to attempted suicide recorded at a toxicological information and assistance center in Fortaleza, Ceará, Brazil, in 2022.

**Methods:**

Cross-sectional study with data recorded from January 1st to December 31, 2022. The population included all cases of poisoning due to attempted suicide registered by the center during the period. The data were collected from the Brazilian Poisoning Data System of the Toxicological Information and Assistance Centers. The analysis was based on descriptive statistics, through the calculation of absolute and relative frequencies for each variable.

**Results:**

360 cases of attempted suicide were identified, with the main groups of toxic agents being medications (64.4%), pesticides (18.9%) and combinations of agents (10.3%). Most of the cases involved females (61.4%), occurred in urban areas (91.1%), in Fortaleza (65.3%) and in homes (88.0%). The age group of 20-29 years (29.3%) with ‘student’ as the occupation (15.6%) stood out among the records. In 90.3% of cases, there were clinical signs and symptoms, and the oral route was the most used (97.5%); 45.5% required hospitalization; and 79.4% recovered. Pesticides had the highest lethality (8.8%).

**Conclusion:**

The results showed that suicide attempts by poisoning mainly affect women, occur predominantly at home, and have a favorable clinical outcome. Medications were the most involved toxic agents, and pesticides were the most lethal. These results reinforce the importance of continuous monitoring of cases and the implementation of preventive strategies aimed at the most vulnerable groups.

## Resumen

**Objetivo:**

Delinear el perfil epidemiológico de las intoxicaciones por intento de suicidio registradas en un centro de información y asistencia toxicológica de Fortaleza, Ceará, en 2022.

**Métodos:**

Estudio transversal con datos registrados del 1 de enero al 31 de diciembre de 2022. La población comprendió todos los casos de intoxicación por intento de suicidio atendidos por el centro durante el período. Los datos fueron recolectados del Sistema Brasileño de Datos de Intoxicaciones de los Centros de Información y Asistencia Toxicológica. El análisis se basó en estadística descriptiva, mediante el cálculo de frecuencias absolutas y relativas para cada variable.

**Resultados:**

Se identificaron 360 casos de intento de suicidio, siendo los principales grupos de agentes tóxicos medicamentos (64,4%), pesticidas (18,9%) y combinaciones (10,3%). La mayoría de los accidentes afectaron al sexo femenino (61,4%), ocurrieron en áreas urbanas (91,1%), en Fortaleza (65,3%) y en domicilios (88,0%). Entre los registros sobresalen el grupo de edad de 20 a 29 años (29,3%) y la ocupación estudiante (15,6%). En el 90,3% de los casos hubo signos y síntomas clínicos y la vía oral fue la mayoritaria (97,5%); El 45,5% requirió hospitalización; y el 79,4% se curaron. Los pesticidas tuvieron la mayor letalidad (8,8%).

**Conclusión:**

Los resultados mostraron que los intentos de suicidio por intoxicación afectan principalmente a mujeres, ocurren predominantemente en el hogar y tienen una evolución clínica favorable. Los medicamentos fueron los agentes tóxicos más comúnmente involucrados y los pesticidas los más letales. Estos resultados refuerzan la importancia del seguimiento continuo de los casos y la implementación de estrategias preventivas dirigidas a los grupos más vulnerables.

**Ethical aspects**

This research respected ethical principles, having obtained the following approval data:

Research Ethics Committee: Universidade Federal do Ceará

Opinion number: 5,936,031

Approval date: 10/3/2024

Certificate of Submission for Ethical Appraisal: 67210323.9.0000.5054

Informed Consent Form: Exempted.

## Introduction

Poisoning constitutes a relevant public health problem. In turn, fatal poisoning due to attempted suicide is counted as a case of suicide, being one of the main causes of death globally. Every year, approximately 703,000 deaths by suicide occur worldwide, and 77.0% of these cases are recorded in low- and middle-income countries. It is estimated that for every suicide, there are more than 20 other attempts (1).

Brazil is among the ten countries with the highest number of deaths from this cause (2). Between 2010 and 2019, there were 112,230 deaths by suicide, and the number of deaths increased by 43.0%, rising from 9,454 to 13,523, according to the Brazilian Ministry of Health (3).

In Ceará, suicides increased between 2009 and 2023, mainly among adolescents and young adults. During this period, 9,290 deaths by suicide were recorded, with an average annual rate of 7.0 per 100,000 inhabitants. In 2021, at the height of the SARS-CoV-2 pandemic, the number of deaths by suicide in Ceará increased by 35.8% compared to the previous year. Poisoning was the method used in 13.7% of these cases (4).

Suicidal behavior involves suicidal ideation, attempted suicide, and death by suicide (5), and mental illnesses such as depression and anxiety account for approximately 80.0% of cases (3). The main methods used are hanging, firearms and toxic agents (fatal poisoning) and jumping from great heights (6). The choice involves numerous perspectives, such as access to the method and psychological, emotional, social, and cultural aspects (7). 

Toxic agents are commonly used in self-inflicted violence, such as household cleaning products, drugs of abuse, pesticides, and medications (2). It is estimated, for example, that 15.0% to 20.0% of suicides worldwide occur due to the intentional ingestion of pesticides, generating up to 140,000 deaths per year. (8). 

Given the above, the epidemiological description of the affected local population is essential to understand the situations with highest risk related to suicidal behavior. Health care must be focused on the articulation of strategies for tracking, monitoring, prevention, intervention, and rehabilitation of individuals, contributing to the reduction of deaths (7).

Despite its relevance, there is a lack of studies investigating this issue in depth, which reinforces the relevance of this research. Furthermore, it is expected that this study will serve as a subsidy for the formulation of public policies that aim to mitigate this problem and encourage new research that elucidates the distinct factors associated with suicidal behavior (9).

In this sense, the present study aims to outline the epidemiological profile of cases of poisoning due to attempted suicide registered at a Toxicological Information and Assistance Center, located in Fortaleza (Ceará), in 2022.

## Methods

### Study design

This is a cross-sectional and descriptive study of cases of poisoning due to attempted suicide treated by the Toxicological Information and Assistance Center, in Fortaleza, Ceará.

### Context

This center operates on a 24/7 basis within a tertiary hospital specializing in emergencies and urgent care. The team is made up of doctors, nurses, pharmacists, and administrative staff. Data used in this study refer to the period from January 1^st^ to December 31, 2022.

### Participants

The study population comprised all cases of poisoning due to attempted suicide treated by the center during the aforementioned period, excluding other circumstances of exposure. The classification of these cases as attempted suicide was based on the records completed by the professionals responsible for the care in the Brazilian Poisoning Data System of the Toxicological Information and Assistance Centers.

### Variables 

We organized the characteristics analyzed into four main categories. The first covers aspects related to the patient. The second addresses the characteristics of the toxic agent, including the groups of agents involved in the recorded events. The third category considers the association with exposure to the toxic agent and the poisoning. Finally, the fourth category deals with cases of death from the groups of toxic agents involved, as detailed below:

1. Related to the patient: sex, age group (in complete years), profession/occupation.
2. Related to the toxic agent:
  Groups of toxic agents involved in the recorded events:
  - Medications: classified by therapeutic group, according to the Anatomical, Therapeutic and Chemical Classification (10). It should be noted that the number of medications used in cases of drug poisoning is higher, as a single individual can use two or more medications simultaneously.
  - Pesticides: classified according to the target pest into insecticides, herbicides, and others (11);
  - Household sanitizing products: classified as soaps and detergents, disinfectants, and cleaning agents (12);
  - Drugs of abuse and others;
  - Association (simultaneous use) of chemical substances: two or more, belonging to the same group of toxic agents and also substances from separate groups of toxic agents.
3. Associated with exposure to toxic agents and poisoning: municipality, area, place of exposure, route of exposure, clinical manifestations, need for hospitalization and clinical outcome.
4. Regarding death cases: groups of toxic agents involved, description of the toxic agents involved and determination of lethality for each group.

### Data sources and measurement

The cases seen are recorded in the database of the Brazilian Poisoning Data System of Toxicological Information and Assistance Centers, and the data were collected through the Business Intelligence tool, integrated into this system. This is a computerized virtual access system maintained by the Brazilian Association of Toxicological Information and Assistance Centers, and its purpose is to record, monitor, store, process and recover data from patients seen by the service. The system has a Human Exposure, Animal Exposure, and Information Service Form, in which data on patients treated by the health service are recorded. (13).

### Bias control

To control selection bias, we insured the inclusion of all cases of attempted suicide treated by the center during the aforementioned period. Data recording in this system was conducted by a team trained for this purpose. Incomplete data was flagged as “incomplete” or “unknown.” 

### Study size

The study sample was determined by selecting all cases of attempted suicide treated during the period.

### Statistical methods

Statistical analysis was conducted using Microsoft Excel, version 2019 and EpiInfo, version 7.2.5.0. The analysis was based on descriptive statistics, through the calculation of absolute and relative frequencies for each variable.

To determine the fatality rate, the number of deaths recorded in a given group of toxic agents was divided by the total number of cases of poisoning in that same group, followed by multiplying the quotient by 100.

### Data availability

The research database was stored in the Scientific Electronic Library Online Data repository, with restricted access (14).

## Results

The Ceará Toxicological Information and Assistance Center received 3,824 cases of exposure to toxic agents in 2022, of which 360 (9.4%) occurred due to suicide attempts. The groups of toxic agents most frequently involved in suicide attempts were medications (n=232; 64.4%), pesticides (n=68; 18.9%), household cleaning products (n=20; 5.6%), drugs of abuse (n=1; 0.3%) and agents that were not identified (n=2; 0.6%). There were also reports of poisoning resulting from the simultaneous use (association) of toxic agents belonging to distinct groups (n=37; 10.3%) (Table 1).

**Table 1:** Groups of toxic agents used in suicide attempts, with absolute number of cases and relative percentage within the specified group. Fortaleza, 2022 (n=360)

Groups of toxic agents	n (%)
**Medications (ATC code)^a^**	**232** (64.4)
Antidepressants (N06A)	111 (22.0)
Antiepileptics (N03A)	101 (20.0)
Antipsychotics (N05A)	60 (11.9)
Benzodiazepine-derived anxiolytics (N05B)	50 (9.9)
Other analgesics and antipyretics (N02B)	35 (6.9)
Nonsteroidal anti-inflammatory/antirheumatic drugs (M01A)	20 (4.0)
Antihistamines for systemic use (R06A)	20 (4.0)
Glucose-lowering drugs/other than insulin (A10B)	12 (2.4)
Muscle relaxants/centrally acting agents (M03B)	10 (2.0)
Hypnotics and sedatives (N05C)	10 (2.0)
Angiotensin II receptor blockers/plain (C09C)	9 (1.8)
Other classes	67 (13.4)
Pesticides	**68** (18.9)
Pesticides (used in agriculture)	45 (66.2)
Rodenticides	17 (25.0)
Pesticides for veterinary use	3 (4.4)
Pesticides for domestic use	3 (4.4)
**Household cleaning products**	**20** (5.6)
Bleach	10 (50.0)
Muriatic acid	4 (20.0)
Caustic soda	2 (10.0)
Muriatic acid + bleach	1 (5.0)
Muriatic acid + caustic soda	1 (5.0)
Bleach + undetermined disinfectant	1 (5.0)
Common kerosene	1 (5.0)
**Drugs of abuse**	**1** (0.3)
Cocaine	1 (100.0)
**Undetermined agents**	**2** (0.6)
**Associations of agents from separate groups**	**37** (10.3)
Medication + drug of abuse	20 (54.1)
Medication + pesticide for veterinary use	5 (13,5)
Drug of abuse + pesticide (used in agriculture)	2 (5.4)
Medication + pesticide (used in agriculture)	3 (8,1)
Drug of abuse + rodenticide	1 (2.7)
Medication + food supplement	1 (2.7)
Medication + rodenticide	1 (2.7)
Medication + rodenticide + pesticide (used in agriculture)	1 (2.7)
Medication + household cleaning product	1 (2.7)
Household cleaning agent + drug of abuse	1 (2.7)
Other (petrol + glass)	1 (2.7)

^a^ Anatomical, Therapeutic and Chemical Classification (10).

Medications were the most relevant (n=232; 64.4%). The most frequent classes were psychotropic drugs (n=323; 63.8%), such as antidepressants, antiepileptics, antipsychotics and benzodiazepines. A single active ingredient was involved in 108 cases (46.5%), two in 54 (23.3%), three in 23 (9.9%) and four or more active ingredients in 47 (20.2%) cases. In total, 505 (100.0%) medications were used, and the most commonly used were amitriptyline and clonazepam (n=41; 8.1% each), valproic acid and diazepam (n=22; 4.3% each), alprazolam and carbamazepine (n=20; 4.0% each), paracetamol (n=17; 3.4%), dipyrone and fluoxetine (n=15; 3.0% each), sertraline (n=14; 2.8%) and others (n=278; 55.0%) (Table 1).

Among pesticides, the majority involved agrochemicals (intended for agricultural use) (n=45; 66.2%), followed by rodenticides (n=17; 25.0%) and pesticides for veterinary and domestic use (n=3; 4.4% each). “Chumbinho” (granular carbamate rodenticide), banned in Brazil, was the most commonly used agricultural product (n=15; 33.0%). Among those for domestic and veterinary use, dichlorvos, chlorpyrifos and cypermethrin were the most used (n=6; 100.0%). Finally, the most commonly used rodenticide was brodifacoum (n=8; 47.1%) (Table 1).

In cases of exposure to household cleaning products, bleach was the most common agent (50.0%), followed by muriatic acid (20.0%), caustic soda (10.0%) and common kerosene (5.0%). In 15.0% (n=3) of cases, there was a combined ingestion of different agents, such as muriatic acid and bleach (Table 1). 

There was a sole case of cocaine exposure (n=1; 0.3%). The toxic agent was not identified in two cases (n=2; 0.6%). The simultaneous use of substances belonging to distinct groups of toxic agents was also recorded (n=37; 10.3%) (Table 1).

The majority of individuals were female (n=221; 61.4%). However, exposure to pesticides (n=39; 57.4%) and multiple toxic agents (n=21; 56.8%) occurred mainly among men. The most affected age group was between 15 and 49 years old (n=293; 81.4%), and the most frequent occupation was student (n=56; 15.6%). Most exposures occurred in urban areas (n=328; 91.1%), mainly in Fortaleza (n=235; 65.3%) and in the usual residential environment (n=317; 88.1%) (Table 2).

**Table 2:** Distribution of the variables: sex, age group, patient’s occupation, municipality, zone, and place of occurrence of exposure to the toxic agent, with absolute number of cases and relative percentage within the specified group. Fortaleza, 2022 (n=360)

Variables	Medications		Pesticides	Household cleaning products	Drugs of abuse	Associations	Undetermined agents	Total
	n (%)		n (%)	n (%)	n (%)	n (%)	n (%)	n (%)
Gender								
Male	67 (28.9)		39 (57.4)	10 (50.0)	1 (100.0)	21 (56.8)	1 (50.0)	139 (38.6)
Female	165 (71.1)		29 (42.7)	10 (50.0)	-	16 (43.2)	1 (50.0)	221 (61.4)
**Age group** (years)								
5-9	1 (0.4)		-	-	-	-	-	1 (0.3)
10-14	22 (9.5)		-	-	-	-	-	22 (6.1)
15-19	51 (22.0)		7 (10.3)	4 (20.0)	-	4 (10,8)	1 (50.0)	67 (18.6)
20-29	66 (28.5)		20 (29.4)	3 (15.0)	-	13 (35.1)	-	102 (28.3)
30-39	47 (20.3)		16 (23.5)	3 (15.0)	1 (100.0)	10 (27.0)	-	77 (21.4)
40-49	27 (11.6)		12 (17.7)	2 (10.0)	-	6 (16.2)	-	47 (13.1)
50-59	14 (6.0)		9 (13.2)	4 (20.0)	-	4 (10,8)	1 (50.0)	32 (8.9)
60-69	3 (1,3)		1 (1.5)	1 (5.0)	-	-	-	5 (1.4)
70-79	1 (0.4)		1 (1.5)	2 (10.0)	-	-	-	4 (1.1)
≥80	-		2 (2.9)	1 (5.0)	-	-	-	3 (0,8)
Profession/occupation								
Student	50 (21.6)		2 (2.9)	3 (15.0)	-	1 (2.7)	-	56 (15.6)
Unemployed	16 (6.9)		3 (4.4)	1 (5.0)	-	3 (8,1)	1 (50.0)	24 (6.7)
Homemaker	10 (4.3)		2 (2.9)	1 (5.0)	-	3 (8,1)	-	16 (4.4)
Unknown	7 (3.0)		4 (5,9)	-	-	2 (5.4)	-	13 (3.6)
Incomplete	124 (53.5)		46 (67.7)	10 (50)	1 (100.0)	21 (56.8)	-	202 (56.1)
Other	25 (10.8)		11 (16.2)	5 (25.0)	-	7 (18.9)	1 (50.0)	49 (13.6)
Municipality								
Fortaleza	154 (66.4)		39 (57.4)	13 (65.0)	-	28 (75.7)	1 (50.0)	235 (65.3)
Caucaia	7 (3.0)		4 (5,9)	-	-	2 (5.4)	-	13 (3.6)
Maranguape	8 (3,5)		2 (2.9)	-	-	1 (2.7)	-	11 (3.1)
Boa Viagem	3 (1,3)		3 (4.4)	-	-	-	-	6 (1.7)
Eusébio	3 (1,3)		2 (2.9)	1 (5.0)	-	-	-	6 (1.7)
Maracanaú	4 (1,7)		-	2 (10.0)	-	-	-	6 (1.7)
Other	53 (22.8)		18 (26.5)	4 (20.0)	1 (100.0)	6 (16.2)	1 (50.0)	83 (23.1)
Zone								
Urban	215 (92.7)		58 (85.3)	19 (95.0)	1 (100.0)	33 (89.2)	2 (100.0)	328 (91.1)
Rural	16 (6.9)		7 (10.3)	1 (5.0)	-	3 (8,1)	-	27 (7.5)
Periurban	-		-	-	-	1 (2.7)	-	1 (0.3)
Unknown	1 (0.4)		3 (4.4)	-	-	-	-	4 (1.1)
Location								
Residence – habitual	214 (92.2)		54 (79.4)	17 (85.0)	1 (100.0)	29 (78.4)	2 (100.0)	317 (88.1)
Residence – other	5 (2.2)		2 (2.9)	1 (5.0)	-	2 (5.4)	-	10 (2.8)
External/public environment	3 (1,3)		2 (2.9)	-	-	1 (2.7)	-	6 (1.7)
Workplace	1 (0.4)		2 (2.9)	1 (5.0)	-	1 (2.7)	-	5 (1.4)
School/nursery	1 (0.4)		-	1 (5.0)	-	-	-	2 (0.6)
Unknown	7 (3.0)		7 (10.3)	-	-	4 (10,8)	-	18 (5.0)
Other	1 (0.4)		1 (1.5)	-	-	-	-	2 (0.6)

The oral route was the most common (n=351; 97.5%), and most cases manifested clinical signs and symptoms (n=325; 90.3%), did not require hospitalization (53.1%) and had total recovery as the clinical outcome (79.4%) (table 3).

**Table 3:** Distribution of variables: route of exposure, clinical manifestations, hospitalization, and clinical outcome, with absolute number of cases and relative percentage within the specified group. Fortaleza, 2022 (n=360)

Variables	Medications		Pesticides	Household cleaning products	Drugs of abuse	Associations	Undetermined agents	Total
	n (%)		n (%)	n (%)	n (%)	n (%)	n (%)	n (%)
**Route of exposure**								
Oral	232 (100.0)		68 (100.0)	20 (100.0)	-	29 (78.4)	2 (100.0)	351 (97.5)
Nasal	-		-	-	1 (100.0)	-	-	1 (0.3)
Oral/nasal	-		-	-	-	8 (21,6)	-	8 (2.2)
**Clinical manifestations**								
Yes	209 (90.1)		59 (86.8)	20 (100.0)	1 (100.0)	34 (91.9)	2 (100.0)	325 (90.3)
No	23 (9.9)		9 (13.2)	-	-	3 (8,1)	-	35 (9.7)
Hospitalization								
Yes	98 (42.2)		39 (57.4)	8 (40.0)	1 (100.0)	17 (46.0)	1 (50.0)	164 (45.6)
No	131 (56.5)		28 (41.2)	11 (55.0)	-	20 (54.1)	1 (50.0)	191 (53.1)
Unknown	3 (1,3)		1 (1.5)	1 (5.0)	-	-	-	5 (1.4)
**Clinical outcome**								
Recovery	193 (83.2)		48 (70.6)	13 (65.0)	1 (100.0)	30 (81.1)	1 (50.0)	286 (79.4)
Asymptomatic	3 (1,3)		7 (10.3)	-	-	3 (8,1)	1 (50.0)	14 (3.9)
Event-related death	5 (2.2)		6 (8.8)	2 (10.0)	-	-	-	13 (3.6)
Probable recovery	4 (1,7)		-	1 (5.0)	-	1 (2.7)	-	6 (1.7)
Sequel	1 (0.4)		1 (1.5)	1 (5.0)	-	-	-	3 (0,8)
Differential diagnosis	1 (0.4)		1 (1.5)	-	-	-	-	2 (0.6)
Death from another cause	2 (0.9)		-	-	-	-	-	2 (0.6)
Unknown	23 (9.9)		5 (7,4)	3 (15.0)	-	3 (8,1)	-	34 (9.4)

The fatality rate was 4.16% (n=15). Among the deaths, seven were caused by medications, six by pesticides and two by household cleaning products. Exposure to sanitizing agents presented the highest mortality (10.0%), and muriatic acid was the main representative. Pesticides had the second highest lethality (8.8%), with emphasis on anticholinesterase insecticides (carbamate or organophosphate), the herbicides 2,4-dichlorophenoxyacetic acid and glyphosate (N-phosphonomethyl glycine) and another unidentified herbicide. Medications had the lowest lethality (3.0%), and the main drugs involved were psychotropic drugs (Table 4).

**Table 4:** Distribution of toxic agents involved in cases that resulted in death, with absolute number of cases (n) within the specified group. Fortaleza, 2022 (n=15)

Toxic agent	Group of toxic agents	n
Anticholinesterase insecticide (carbamate or organophosphate)	Pesticide	4
Herbicide 2,4-D	Pesticide	1
Combination of glyphosate herbicide and another undetermined herbicide	Pesticides	1
Hydrochloric acid (active ingredient in muriatic acid)	Household cleaning agent	2
Valproic acid	Medication	1
Association of valproic acid and zolpidem	Medications	1
Combination of alprazolam, amitriptyline, bisacodyl, caffeine, chlordiazepoxide, duloxetine, norethisterone, olanzapine and paracetamol	Medications	1
Combination of amitriptyline, diazepam, fluoxetine, haloperidol, and promethazine	Medications	1
Combination of bupropion, lithium carbonate, clonazepam, mirtazapine, olanzapine, and venlafaxine	Medications	1
Combination of carbamazepine and quetiapine	Medications	1
Combination of citalopram, chlorpromazine, fluoxetine, nortriptyline, and promethazine	Medications	1

## Discussion

Medications were the main agents implicated in the cases, followed by pesticides and household cleaning products. The substantial contribution of the simultaneous use of substances from separate groups of toxic agents in these cases stands out. Most involved females and people aged between 15 and 49 years old. The most frequent professions/occupations were the following: student, unemployed and homemaker. The cases occurred mainly in the urban area of Fortaleza and in the usual residential environment. The oral route was the most used. In most cases, clinical signs and symptoms developed without the need for hospital admission, and recovery was the clinical outcome. Household sanitizing products had the highest fatality rate, followed by pesticides and medications. The deaths were caused by pesticides (anticholinesterase insecticides and 2,4-dichlorophenoxyacetic and glyphosate herbicides), household cleaning products (hydrochloric acid) and medications (valproic acid and combinations of several psychoactive medications).

The study has limitations, such as the cross-sectional analysis based on secondary data and the high number of unknown or incomplete fields, revealing flaws in the registration. Furthermore, the data were collected from a single health unit in Ceará, reflecting only the reality of individuals who received assistance after exposure to toxic agents. It is also worth noting the possible underreporting of attempted suicide cases, since not all of them are treated by a health unit. Finally, the absence of clinical and psychosocial data on patients limits an in-depth understanding of the context of these occurrences.

It was observed that the group of toxic agents most involved in suicide attempts was medication. This finding agrees with the results obtained by studies on the epidemiological profile of attempted suicide by poisoning conducted in Piauí, in 2020 (15), and in Espírito Santo, in 2019 (16).

The high predominance of medications occurs because they are probably, in general, freely accessible, low cost and socially accepted (2). Furthermore, the massive advertising by the pharmaceutical industry and the habit of stockpiling them at home make medications one of the most readily available means for use in attempts at self-extermination (17).

The main classes of mediations identified were those used to treat psychiatric disorders, either in contexts of attempted self-extermination through drug poisoning (17-19) or through developed poisoning (17). This possibly happens because most cases of suicidal behavior are related to mental disorders and psychological suffering, the strategy for which also involves drug therapy with psychotropic drugs (18).

Therefore, controlling the sale of medications, correctly disposing of those that are no longer in use or have expired, discouraging storage and frequent monitoring of patients using psychotropic drugs by the health team are useful strategies to reduce the use of medications in these cases (20).

The second most frequent group of toxic agents among the records was pesticides. Brazil, one of the largest agricultural producers in the world, has been the leader in pesticide consumption since 2008. Furthermore, from 2010 to 2020, there was a 78.3% increase in the amount of pesticides sold in the country (21). 

The availability of these products in rural areas makes them an easy choice as a means of self-extermination. Due to significant national consumption, the legal acquisition of pesticides is facilitated, making them more available. The population’s knowledge about the high lethality of these substances also encourages their use in self-inflicted violence (22).

In this scenario, it is essential to adopt measures that restrict access to pesticides. For example, in the 1990s, Sri Lanka had one of the highest suicide rates in the world. The country’s gradual elimination of pesticides reduced this rate by around 70% over 20 years, saving around 93,000 lives (8). 

Household cleaning products were the fourth most commonly involved group of toxic agents among those recorded. In an epidemiological study on suicide attempts using toxic agents conducted at the same center, with data from 2017 and 2018, sanitizing agents were also the fourth most frequent group (23). The wide variety of these products in supermarkets and their presence in people’s homes and daily lives increase the risk of poisoning and their use in suicide attempts (5).

Females were reported more frequently in these cases, a finding that is in line with the results of studies on suicide attempts due to the use of toxic agents in Piauí (15), Espírito Santo (16) and Ceará (23). Suicidal behavior among women can be attributed to several factors, such as overload of family responsibilities, exposure to domestic violence, gender inequality and pressures imposed by patriarchy (23).

The toxic agents most used by women were medications, probably due to the tendency to choose less aggressive methods, such as toxic substances. On the other hand, men prefer more violent methods, such as hanging or firearms. The choice of method based on gender reflects its availability and social acceptability (23). Thus, the ingestion of medication by women would be more socially accepted than for men, from whom more aggressive methods are expected by society, although medications are equally available to both sexes (24).

The age group most affected was 20 to 39 years old. The greater involvement of adults in cases of attempted suicide was also observed in Piauí (15) and Espírito Santos (16). In fact, this phase of life can be marked by vulnerabilities, such as financial difficulties, problems at work, social and family conflicts, abuse of illicit substances and psychiatric disorders, factors that influence suicidal behavior (25).

A considerable number of adolescents (age range 15–19 years) were also identified. This probably occurs because this moment in life is marked by biopsychosocial instabilities, such as family demands, academic changes, and insecurity about the future (15,25). 

The student occupation was the most reported, a finding in agreement with the results of two other epidemiological studies on self-extermination by poisoning (20,23). This situation may be related to academic pressure, social isolation, abuse of psychoactive substances and psychological illnesses that can characterize the school period (15). The second most registered occupation was that of unemployed people. It is noteworthy that unemployed men are more predisposed to suicide attempts, possibly due to increased financial stress and lack of professional prospects (25).

Most of the patients involved lived in the urban area, in the metropolitan region of Fortaleza. This can be explained by the fact that the only center specialized in caring for individuals exposed to toxic agents in Ceará is located in Fortaleza, which facilitates access to care for residents of the capital and surrounding areas, compared to residents of more distant areas (26).

Exposure to toxic agents occurred mainly in the usual residential environment, highlighting the role of family relationships as a risk factor for suicidal behavior, which may include parental conflicts, marital crises, and financial difficulties (26).

Although clinical manifestations occurred in most cases of exposure to toxic agents, the results obtained showed a favorable clinical outcome, with a predominance of recovery for all groups of toxic agents. The potential for acute toxicity of the toxic agent may be related to the occurrence of clinical manifestations, the need for hospitalization of the victim and the outcome. Thus, pesticides, known for their significant acute toxicity, had a higher hospitalization rate compared to the other groups. It should be considered that the severity of the clinical condition also involves the use of multiple substances, the quantity ingested, age, previous history and the time elapsed between exposure and treatment (26).

Household cleaning products had the highest fatality rate, and hydrochloric acid, the active ingredient in muriatic acid, was responsible for the deaths that occurred. This strong acid causes tissue destruction through coagulation necrosis and free radical formation, and the extent of the damage depends on the concentration of the formulation, the duration of contact, the amount ingested and the presence or absence of food in the stomach (27).

Anticholinesterase insecticides, such as organophosphates and carbamates, are responsible for the highest number of deaths from pesticides (used in agriculture) (12). They inhibit acetylcholinesterase, an enzyme responsible for degrading acetylcholine, which causes hyperstimulation of cholinergic receptors, generating cholinergic syndrome. This syndrome includes excessive sweating, salivation, bronchoconstriction, increased gastrointestinal motility, tremors, among other effects. Atropine is the antidote used in the treatment of acute poisoning (11).

At the Ceará Toxicological Information and Assistance Center, the use of anticholinesterase is identified based on information provided by the patient or companion, the signs and symptoms manifested and the determination of the inhibition of the activity of the enzyme butyrylcholinesterase in the plasma, which serves as a biological indicator of exposure to these insecticides (11).

Deaths were also caused by the herbicide 2,4-dichlorophenoxyacetic acid. When ingested in massive quantities, the patient commonly presents phenolic odor, headache, gastrointestinal disorders, hemorrhage, neuromuscular changes, lethargy, torpor, and coma in the most severe cases (12). There is no specific antidote for this herbicide, which in 2015 was classified by the International Agency for Research on Cancer as possibly carcinogenic to humans (11).

Glyphosate is a systemic, non-selective herbicide with low acute toxicity, used to control annual and perennial plants. The most widely used glyphosate-based product is Roundup, whose formulation uses polyoxethyleneamine as a surfactant. It has been reported that this substance can increase cellular permeability to glyphosate (11). 

Acute poisoning resulting from exposure to glyphosate results in mild gastrointestinal symptoms, while moderate or severe poisoning manifests with gastrointestinal bleeding, hypotension, pulmonary dysfunction, and kidney damage. There is no specific antidote for this herbicide (12), classified by the International Agency for Research on Cancer as a probable carcinogen for humans in 2015 (11).

Medications presented the lowest fatality rate among the groups studied, which is compatible with the fact that only one of the cases of death was caused by a single agent, valproic acid. All others were due to the simultaneous ingestion of multiple medications. The effect of the combined action of several agents leads to the occurrence of more severe poisonings, including fatal ones, as observed in the cases evaluated (11).

It is worth highlighting the role of primary healthcare in identifying, evaluating, managing, and referring cases related to suicidal behavior, developing actions aimed at early identification and timely intervention. Its integration with mental health services strengthens psychosocial support and ensures adequate monitoring of these cases. Access to specialized treatments, combined with mental health support networks, helps reduce psychological suffering and prevent new suicide attempts (28).

By presenting the profile of individuals and toxic agents involved in suicide attempts recorded at the Ceará Toxicological Information and Assistance Center, this study contributes with the knowledge needed to guide policies and measures to prevent these occurrences. Among them, the following stand out: regulating access to toxic agents, strengthening psychological support programs, and expanding awareness campaigns for suicide prevention. Thus, the study contributes to reducing suicide attempts and protecting the identified vulnerable populations.

## Data Availability

The database and analysis codes used in the research are available at: https://data.scielo.org/dataset.xhtml?persistentId=doi:10.48331/scielodata.CAI7TX.
