# Supplementary material for: Suicide attempts by poisoning recorded at a toxicological information and assistance center: a cross-sectional study, Fortaleza, 2022
Source: Epidemiol Serv Saude. 2025 Aug 11;34:e20240885. doi: 10.1590/S2237-96222025v34e20240885.en (PMC12342851; doi:10.1590/S2237-96222025v34e20240885.en)
Supplement: Translation

# Translation: Tentativas de suicídio por intoxicação registradas em um centro de informação e assistência toxicológica: estudo transversal, Fortaleza, 2022

**DOI:** 10.1590/S2237-96222025v34e20240885.pt

**Authors:** Hendyelle Rodrigues Ferreira e Silva, Wanderley Pinheiro de Holanda, Karla do Nascimento Magalhães, Dário Luis do Nascimento Magalhães, Alan Queiroz de Souza Santos, Maria Augusta Drago Ferreira

**Affiliations:** 1 Escola de Saúde Pública do Ceará, Residência Multiprofissional em Urgência e Emergência, Fortaleza, CE, Brasil; 2 Perícia Forense do Estado do Ceará, Núcleo de Toxicologia Forense, Fortaleza, CE, Brasil; 3 Instituto Doutor José Frota, Centro de Informação e Assistência Toxicológica, Fortaleza, CE, Brasil; 4 Universidade Estadual do Ceará, Centro de Ciências da Saúde, Fortaleza, CE, Brasil; 5 Universidade Federal do Ceará, Departamento de Farmácia, Fortaleza, CE, Brasil

**Keywords:** Ideação Suicida, Intoxicação, Toxicologia, Epidemiologia, Análise Transversal

## Abstract

**Objetivo:**

Traçar o perfil epidemiológico das intoxicações por tentativa de suicídio registrados em um centro de informação e assistência toxicológica em Fortaleza, Ceará, em 2022.

**Métodos:**

Estudo transversal com dados registrados no período de 1o de janeiro a 31 de dezembro de 2022. A população compreendeu todos os casos de intoxicação por tentativa de suicídio atendidos pelo centro no período. Os dados foram coletados a partir do Sistema Brasileiro de Dados de Intoxicações dos Centros de Informação e Assistência Toxicológica. A análise se apoiou em estatística descritiva, por meio do cálculo das frequências absolutas e relativas para cada variável.

**Resultados:**

360 casos de tentativa de suicídio foram identificados, sendo os principais grupos de agentes tóxicos os medicamentos (64,4%), os praguicidas (18,9%) e as associações (10,3%). A maioria envolveu o sexo feminino (61,4%), ocorreu na zona urbana (91,1%), em Fortaleza (65,3%) e nas residências (88,0%). A faixa etária de 20-29 anos (29,3%) e a ocupação de estudante (15,6%) destacaram-se entre os registros. Em 90,3% dos casos, houve sinais e sintomas clínicos, e a via oral foi a majoritária (97,5%); 45,5% necessitaram de internação; e 79,4% foram curados. Os praguicidas tiveram a maior letalidade (8,8%).

**Conclusão:**

Os resultados evidenciaram que a tentativa de suicídio por intoxicação acomete majoritariamente mulheres, ocorre predominantemente no domicílio e tem desfecho clínico favorável. Os medicamentos foram os agentes tóxicos mais envolvidos, e os praguicidas apresentaram maior letalidade. Esses resultados reforçam a importância do monitoramento contínuo dos casos e da implementação de estratégias preventivas direcionadas aos grupos mais vulneráveis.

**Aspectos éticos**

A presente pesquisa respeitou os princípios éticos, obtendo os seguintes dados de aprovação:

Comitê de ética em pesquisa: Universidade Federal do Ceará

Número do parecer: 5.936.031

Data de aprovação: 10/3/2024

Certificado de apresentação de apreciação ética: 67210323.9.0000.5054

Registro do consentimento livre e esclarecido: Dispensado.

## Introdução

A intoxicação constitui um relevante problema de saúde pública. Por sua vez, as intoxicações fatais por tentativa de suicídio são contabilizadas como casos de suicídio, sendo uma das principais causas de morte em nível global. Anualmente, ocorrem cerca de 703 mil óbitos por suicídio no mundo, e 77,0% desses casos são registrados nos países de baixa e média renda. Estima-se que, para cada suicídio, há mais de 20 outras tentativas (1).

O Brasil está entre os dez países com o maior número de óbitos por essa causa (2). Entre 2010 e 2019, ocorreram 112.230 óbitos por suicídio, e o número de mortes aumentou 43,0%, passando de 9.454 para 13.523, segundo o Ministério da Saúde (3).

No Ceará, o suicídio aumentou entre 2009 e 2023, principalmente entre adolescentes e jovens adultos. Nesse período, foram registrados 9.290 óbitos por suicídio, com uma taxa média anual de 7,0 por 100 mil habitantes. Em 2021, no auge da pandemia de SARS-CoV-2, o número de óbitos por suicídio no Ceará aumentou 35,8% em relação ao ano anterior. A intoxicação foi o método utilizado em 13,7% desses casos (4).

O comportamento suicida envolve a ideação suicida, a tentativa de suicídio e a morte por suicídio (5), e doenças mentais, como depressão e ansiedade, representam cerca de 80,0% dos casos (3). Os principais métodos empregados são o enforcamento, o emprego de arma de fogo e de agentes tóxicos (intoxicação fatal) e o salto de grandes alturas (6). A escolha envolve inúmeras perspectivas, como a disponibilidade de acesso ao método e os aspectos psicológicos, emocionais, sociais e culturais (7). 

Agentes tóxicos comumente são empregados nas violências autoinfligidas, como saneantes domissanitários, drogas de abuso, praguicidas e medicamentos (2). Por exemplo, estima-se que de 15,0% a 20,0% dos suicídios, em todo o mundo, acontecem por ingestão intencional de praguicidas, gerando até 140 mil mortes por ano (8). 

Dado o exposto, a descrição epidemiológica da população local acometida é fundamental para compreender as situações de maior risco relacionadas ao comportamento suicida. A atenção à saúde deve estar focada na articulação de estratégias de rastreamento, monitoramento, prevenção, intervenção e reabilitação dos indivíduos, contribuindo para a redução de óbitos (7).

Apesar da relevância, nota-se a carência de estudos que investiguem com profundidade tal questão, o que reforça a pertinência desta pesquisa. Além disso, espera-se que este estudo sirva como subsídio para a formulação de políticas públicas que visem mitigar tal problemática, e incentive a realização de novas pesquisas que elucidem os diferentes fatores associados ao comportamento suicida (9).

Nesse sentido, o presente estudo objetiva traçar o perfil epidemiológico dos casos de intoxicação por tentativa de suicídio registrados em um Centro de Informação e Assistência Toxicológica, localizado em Fortaleza (Ceará), no ano de 2022.

## Métodos

### Delineamento

Trata-se de um estudo transversal e descritivo dos casos de intoxicação por tentativa de suicídio atendidos pelo Centro de Informação e Assistência Toxicológica, em Fortaleza, Ceará.

### Contexto

Esse centro funciona de forma ininterrupta dentro de um hospital de nível terciário especializado em urgência e emergência. A equipe de trabalho é composta por médicos, enfermeiros, farmacêuticos e funcionários administrativos. Os dados são referentes ao período de 1^o^ de janeiro a 31 de dezembro de 2022.

### Participantes

A população do estudo compreendeu todos os casos de intoxicação por tentativa de suicídio atendidos pelo centro no referido período, sendo excluídas outras circunstâncias de exposição. A classificação desses casos como tentativa de suicídio foi feita com base nos registros preenchidos pelos profissionais responsáveis pelo atendimento no Sistema Brasileiro de Dados de Intoxicações dos Centros de Informação e Assistência Toxicológica.

### Variáveis 

As características analisadas foram organizadas em quatro categorias principais. A primeira abrange os aspectos relacionados ao paciente. A segunda aborda as características do agente tóxico, incluindo os grupos de agentes envolvidos nos eventos registrados. A terceira considera a associação à exposição ao agente tóxico e à intoxicação. Por fim, a quarta categoria trata dos casos de óbito dos grupos de agentes tóxicos implicados, conforme detalhado a seguir:

1. Relacionadas ao paciente: sexo, faixa etária (em anos completos), profissão/ocupação.
2. Relativas ao agente tóxico:
  Grupos de agentes tóxicos envolvidos nos eventos registrados:
  - Medicamentos: classificados por grupo terapêutico, segundo a Classificação Anatômica, Terapêutica e Química (10). Ressalta-se que o número de medicamentos empregados dentro dos casos de intoxicação medicamentosa é superior, pois um único indivíduo pode utilizar dois ou mais medicamentos simultaneamente.
  - Praguicidas: classificados segundo a praga-alvo em inseticidas, herbicidas e outros (11);
  - Saneantes domissanitários: classificados em sabões e detergentes, desinfetantes e agentes de limpeza (12);
  - Drogas de abuso e outros;
  - Associação (uso simultâneo) de substâncias químicas: duas ou mais, pertencentes ao mesmo grupo de agentes tóxicos e, também, de substâncias de grupos diferentes de agentes tóxicos.
3. Associadas à exposição ao agente tóxico e à intoxicação: município, zona, local de ocorrência da exposição, via de exposição, manifestações clínicas, necessidade de internação e desfecho clínico.
4. Referentes aos casos de óbito: grupos de agentes tóxicos envolvidos, descrição dos agentes tóxicos implicados e determinação da letalidade para cada grupo.

### Fontes de dados e mensuração

Os casos atendidos são registrados no banco de dados do Sistema Brasileiro de Dados de Intoxicações dos Centros de Informação e Assistência Toxicológica, e os dados foram coletados por meio da ferramenta Business Intelligence, integrada a esse sistema. Trata-se de um sistema informatizado de acesso virtual mantido pela Associação Brasileira de Centros de Informação e Assistência Toxicológica, e tem por finalidade o registro, acompanhamento, armazenamento, processamento e recuperação dos dados de pacientes atendidos pelo serviço. O sistema dispõe de uma Ficha de Atendimento de Exposição Humana, Exposição Animal e de Informação, na qual são registrados os dados dos pacientes atendidos pelo serviço de saúde (13).

### Controle de viés

Para controle de viés de seleção, garantiu-se a inclusão de todos os casos de tentativa de suicídio atendidos pelo centro no referido período. O registro dos dados nesse sistema foi realizado por equipe treinada para este fim. Os dados preenchidos de forma incompleta foram sinalizados como “não preenchido” ou “ignorado”. 

### Tamanho do estudo

A amostra do estudo foi determinada por meio da seleção de todos os casos de tentativa de suicídio atendidos no período.

### Métodos estatísticos

A análise estatística foi conduzida utilizando os programas Microsoft Excel, versão 2019 e EpiInfo, versão 7.2.5.0. A análise apoiou-se em estatística descritiva, por meio do cálculo das frequências absolutas e relativas para cada variável.

Para determinar o percentual de letalidade, dividiu-se o número de óbitos registrados em um determinado grupo de agente tóxico pelo número total de casos de intoxicações desse mesmo grupo, seguido da multiplicação do quociente por 100.

### Disponibilidade de dados

O banco de dados da pesquisa foi depositado no repositório Scientific Electronic Library Online Data, com acesso restrito (14).

## Resultados

O Centro de Informação e Assistência Toxicológica do Ceará atendeu 3.824 casos de exposição a agentes tóxicos em 2022, dos quais 360 (9,4%) ocorreram devido a tentativas de suicídio. Os grupos de agentes tóxicos envolvidos com maior frequência nas tentativas de suicídio foram medicamentos (n=232; 64,4%), praguicidas (n=68; 18,9%), saneantes domissanitários (n=20; 5,6%), drogas de abuso (n=1; 0,3%) e agentes que não foram identificados (n=2; 0,6%). Houve também relatos de intoxicação decorrentes do uso simultâneo (associação) de agente tóxicos pertencentes a diferentes grupos (n=37; 10,3%) (Tabela 1).

**Tabela 1:** Grupos de agentes tóxicos usados ​​em tentativas de suicídio, com número absoluto de casos e porcentagem relativa dentro do grupo especificado. Fortaleza, 2022 (n=360)

Grupos de agentes tóxicos	n (%)
**Medicamentos** (**código ATC**)^a^	**232** (64,4)
Antidepressivos (N06A)	111 (22,0)
Antiepiléticos (N03A)	101 (20,0)
Antipsicóticos (N05A)	60 (11,9)
Ansiolíticos derivados de benzodiazepínicos (N05B)	50 (9,9)
Outros analgésicos e antipiréticos (N02B)	35 (6,9)
Anti-inflamatórios/antirreumáticos não esteroides (M01A)	20 (4,0)
Anti-histamínicos de uso sistêmico (R06A)	20 (4,0)
Medicamentos para reduzir a glicose/exceto insulina (A10B)	12 (2,4)
Relaxantes musculares/agentes de ação central (M03B)	10 (2,0)
Hipnóticos e sedativos (N05C)	10 (2,0)
Bloqueadores dos receptores da angiotensina II/simples (C09C)	9 (1,8)
Outras classes	67 (13,4)
Praguicidas	**68** (18,9)
Agrotóxicos (praguicidas de uso agrícola)	45 (66,2)
Raticidas	17 (25,0)
Praguicidas de uso veterinário	3 (4,4)
Praguicidas de uso doméstico	3 (4,4)
**Saneantes domissanitários**	**20** (5,6)
Água sanitária	10 (50,0)
Ácido muriático	4 (20,0)
Soda cáustica	2 (10,0)
Ácido muriático + água sanitária	1 (5,0)
Ácido muriático + soda cáustica	1 (5,0)
Água sanitária + desinfetante não determinado	1 (5,0)
Querosene comum	1 (5,0)
**Drogas de abuso**	**1** (0,3)
Cocaína	1 (100,0)
**Agentes não determinados**	**2** (0,6)
**Associações de agentes de diferentes grupos**	**37** (10,3)
Medicamento + droga de abuso	20 (54,1)
Medicamento + praguicida de uso veterinário	5 (13,5)
Droga de abuso + agrotóxico (praguicida de uso agrícola)	2 (5,4)
Medicamento + agrotóxico (praguicida de uso agrícola)	3 (8,1)
Droga de abuso + raticida	1 (2,7)
Medicamento + suplemento alimentar	1 (2,7)
Medicamento + raticida	1 (2,7)
Medicamento + raticida + agrotóxico (praguicida de uso agrícola)	1 (2,7)
Medicamento + saneante domissanitário	1 (2,7)
Saneante domissanitário + droga de abuso	1 (2,7)
Outra (gasolina + vidro)	1 (2,7)

^a^ Classificação Anatômica, Terapêutica e Química (10).

Os medicamentos foram os mais relevantes (n=232; 64,4%). As classes mais frequentes foram as dos psicofármacos (n=323; 63,8%), como antidepressivos, antiepiléticos, antipsicóticos e benzodiazepínicos. Um único princípio ativo foi envolvido em 108 casos (46,5%), dois em 54 (23,3%), três em 23 (9,9%) e quatro ou mais princípios ativos em 47 (20,2%) casos. No total, foram empregados 505 (100,0%) medicamentos, e os mais implicados foram amitriptilina e clonazepam (n=41; 8,1% cada), ácido valproico e diazepam (n=22; 4,3% cada), alprazolam e carbamazepina (n=20; 4,0% cada), paracetamol (n=17; 3,4%), dipirona e fluoxetina (n=15; 3,0% cada), sertralina (n=14; 2,8%) e outros (n=278; 55,0%) (Tabela 1).

Entre os praguicidas, a maioria envolveu agrotóxicos (destinados ao uso agrícola) (n=45; 66,2%), seguidos por raticidas (n=17; 25,0%) e por praguicidas de uso veterinário e doméstico (n=3; 4,4% cada). O “chumbinho”, proscrito no Brasil, foi o de uso agrícola mais implicado (n=15; 33,0%). Entre os de uso doméstico e veterinário, o diclorvós, clorpirifós e cipermetrina foram os mais utilizados (n=6; 100,0%). Por fim, o raticida mais empregado foi o brodifacum (n=8; 47,1%) (Tabela 1).

Nos casos de exposição aos saneantes domissanitários, a água sanitária foi o agente mais comum (50,0%), seguida por ácido muriático (20,0%), soda cáustica (10,0%) e a querosene comum (5,0%). Em 15,0% (n=3) dos casos, houve ingestão combinada de diferentes agentes, como como ácido muriático e água sanitária (Tabela 1). 

Houve um único caso de exposição à cocaína (n=1; 0,3%). O agente tóxico não foi identificado em dois casos (n=2; 0,6%). O emprego simultâneo de substâncias pertencentes a diferentes grupos de agentes tóxicos também foi registrado (n=37; 10,3%) (Tabela 1).

A maioria dos indivíduos era do sexo feminino (n=221; 61,4%). No entanto, a exposição aos praguicidas (n=39; 57,4%) e a múltiplos agentes tóxicos (n=21; 56,8%) ocorreu, majoritariamente, entre homens. A faixa etária mais acometida foi entre 15 e 49 anos (n=293; 81,4%), e a ocupação mais frequente foi a de estudante (n=56; 15,6%). A maior parte das exposições ocorreu na zona urbana (n=328; 91,1%), principalmente em Fortaleza (n=235; 65,3%) e no ambiente residencial habitual (n=317; 88,1%) (Tabela 2).

**Tabela 2:** Distribuição das variáveis ​​sexo, faixa etária, profissão do paciente, município, zona e local de ocorrência da exposição ao agente tóxico, com número absoluto de casos e porcentagem relativa dentro do grupo especificado. Fortaleza, 2022 (n=360)

Variáveis	Medicamentos		Praguicidas	Saneantes domissanitários	Drogas de abuso	Associações	Agentes não determinados	Total
	n (%)		n (%)	n (%)	n (%)	n (%)	n (%)	n (%)
Sexo								
Masculino	67 (28,9)		39 (57,4)	10 (50,0)	1 (100,0)	21 (56,8)	1 (50,0)	139 (38,6)
Feminino	165 (71,1)		29 (42,7)	10 (50,0)	-	16 (43,2)	1 (50,0)	221 (61,4)
**Faixa etária** (anos)								
5-9	1 (0,4)		-	-	-	-	-	1 (0,3)
10-14	22 (9,5)		-	-	-	-	-	22 (6,1)
15-19	51 (22,0)		7 (10,3)	4 (20,0)	-	4 (10,8)	1 (50,0)	67 (18,6)
20-29	66 (28,5)		20 (29,4)	3 (15,0)	-	13 (35,1)	-	102 (28,3)
30-39	47 (20,3)		16 (23,5)	3 (15,0)	1 (100,0)	10 (27,0)	-	77 (21,4)
40-49	27 (11,6)		12 (17,7)	2 (10,0)	-	6 (16,2)	-	47 (13,1)
50-59	14 (6,0)		9 (13,2)	4 (20,0)	-	4 (10,8)	1 (50,0)	32 (8,9)
60-69	3 (1,3)		1 (1,5)	1 (5,0)	-	-	-	5 (1,4)
70-79	1 (0,4)		1 (1,5)	2 (10,0)	-	-	-	4 (1,1)
≥80	-		2 (2,9)	1 (5,0)	-	-	-	3 (0,8)
Profissão/ocupação								
Estudante	50 (21,6)		2 (2,9)	3 (15,0)	-	1 (2,7)	-	56 (15,6)
Desempregado	16 (6,9)		3 (4,4)	1 (5,0)	-	3 (8,1)	1 (50,0)	24 (6,7)
Do lar	10 (4,3)		2 (2,9)	1 (5,0)	-	3 (8,1)	-	16 (4,4)
Ignorado	7 (3,0)		4 (5,9)	-	-	2 (5,4)	-	13 (3,6)
Não preenchido	124 (53,5)		46 (67,7)	10 (50)	1 (100,0)	21 (56,8)	-	202 (56,1)
Outros	25 (10,8)		11 (16,2)	5 (25,0)	-	7 (18,9)	1 (50,0)	49 (13,6)
Município								
Fortaleza	154 (66,4)		39 (57,4)	13 (65,0)	-	28 (75,7)	1 (50,0)	235 (65,3)
Caucaia	7 (3,0)		4 (5,9)	-	-	2 (5,4)	-	13 (3,6)
Maranguape	8 (3,5)		2 (2,9)	-	-	1 (2,7)	-	11 (3,1)
Boa Viagem	3 (1,3)		3 (4,4)	-	-	-	-	6 (1,7)
Eusébio	3 (1,3)		2 (2,9)	1 (5,0)	-	-	-	6 (1,7)
Maracanaú	4 (1,7)		-	2 (10,0)	-	-	-	6 (1,7)
Outros	53 (22,8)		18 (26,5)	4 (20,0)	1 (100,0)	6 (16,2)	1 (50,0)	83 (23,1)
Zona								
Urbana	215 (92,7)		58 (85,3)	19 (95,0)	1 (100,0)	33 (89,2)	2 (100,0)	328 (91,1)
Rural	16 (6,9)		7 (10,3)	1 (5,0)	-	3 (8,1)	-	27 (7,5)
Periurbana	-		-	-	-	1 (2,7)	-	1 (0,3)
Ignorada	1 (0,4)		3 (4,4)	-	-	-	-	4 (1,1)
Local								
Residência – habitual	214 (92,2)		54 (79,4)	17 (85,0)	1 (100,0)	29 (78,4)	2 (100,0)	317 (88,1)
Residência – outra	5 (2,2)		2 (2,9)	1 (5,0)	-	2 (5,4)	-	10 (2,8)
Ambiente externo/público	3 (1,3)		2 (2,9)	-	-	1 (2,7)	-	6 (1,7)
Local de trabalho	1 (0,4)		2 (2,9)	1 (5,0)	-	1 (2,7)	-	5 (1,4)
Escola/creche	1 (0,4)		-	1 (5,0)	-	-	-	2 (0,6)
Ignorado	7 (3,0)		7 (10,3)	-	-	4 (10,8)	-	18 (5,0)
Outro	1 (0,4)		1 (1,5)	-	-	-	-	2 (0,6)

A via oral foi a mais comum (n=351; 97,5%), e a maioria dos casos manifestou sinais e sintomas clínicos (n=325; 90,3%), não precisou de internação hospitalar (53,1%) e teve a cura como desfecho clínico (79,4%) (tabela 3).

**Tabela 3:** Distribuição das variáveis ​​via de exposição, manifestações clínicas, internação e desfecho clínico, com número absoluto de casos e porcentagem relativa dentro do grupo especificado. Fortaleza, 2022 (n=360)

Variáveis	Medicamentos		Praguicidas	Saneantes domissanitários	Drogas de abuso	Associações	Agentes não determinados	Total
	n (%)		n (%)	n (%)	n (%)	n (%)	n (%)	n (%)
**Via de exposição**								
Oral	232 (100,0)		68 (100,0)	20 (100,0)	-	29 (78,4)	2 (100,0)	351 (97,5)
Nasal	-		-	-	1 (100,0)	-	-	1 (0,3)
Oral/nasal	-		-	-	-	8 (21,6)	-	8 (2,2)
**Manifestações clínicas**								
Sim	209 (90,1)		59 (86,8)	20 (100,0)	1 (100,0)	34 (91,9)	2 (100,0)	325 (90,3)
Não	23 (9,9)		9 (13,2)	-	-	3 (8,1)	-	35 (9,7)
Internação								
Sim	98 (42,2)		39 (57,4)	8 (40,0)	1 (100,0)	17 (46,0)	1 (50,0)	164 (45,6)
Não	131 (56,5)		28 (41,2)	11 (55,0)	-	20 (54,1)	1 (50,0)	191 (53,1)
Ignorado	3 (1,3)		1 (1,5)	1 (5,0)	-	-	-	5 (1,4)
**Desfecho clínico**								
Cura	193 (83,2)		48 (70,6)	13 (65,0)	1 (100,0)	30 (81,1)	1 (50,0)	286 (79,4)
Assintomático	3 (1,3)		7 (10,3)	-	-	3 (8,1)	1 (50,0)	14 (3,9)
Óbito relacionado ao evento	5 (2,2)		6 (8,8)	2 (10,0)	-	-	-	13 (3,6)
Cura provável	4 (1,7)		-	1 (5,0)	-	1 (2,7)	-	6 (1,7)
Sequela	1 (0,4)		1 (1,5)	1 (5,0)	-	-	-	3 (0,8)
Diagnóstico diferencial	1 (0,4)		1 (1,5)	-	-	-	-	2 (0,6)
Óbito por outra causa	2 (0,9)		-	-	-	-	-	2 (0,6)
Ignorado	23 (9,9)		5 (7,4)	3 (15,0)	-	3 (8,1)	-	34 (9,4)

A letalidade foi de 4,16% (n=15). Entre os óbitos, sete foram causados por medicamentos, seis por praguicidas e dois por saneantes domissanitários. A exposição aos saneantes apresentou a maior mortalidade (10,0%), e o ácido muriático foi o principal representante. Os praguicidas tiveram a segunda maior letalidade (8,8%), destacando-se os inseticidas anticolinesterásicos (carbamato ou organofosforado), os herbicidas 2,4-diclorofenoxiacético e glifosato (N-fosfonometil glicina) e um outro não identificado. Os medicamentos tiveram a menor letalidade (3,0%), e os principais fármacos implicados foram os psicotrópicos (Tabela 4).

**Tabela 4:** Distribuição dos agentes tóxicos envolvidos nos casos que evoluíram para o óbito, com número absoluto de casos (n) dentro do grupo especificado. Fortaleza, 2022 (n=15)

Agente tóxico	Grupo de agentes tóxicos	n
Inseticida anticolinesterásico (carbamato ou organofosforado)	Praguicida	4
Herbicida 2,4-D	Praguicida	1
Associação de herbicida glifosato e outro herbicida não determinado	Praguicidas	1
Ácido clorídrico (princípio ativo do ácido muriático)	Saneante domissanitário	2
Ácido valproico	Medicamento	1
Associação de ácido valproico e zolpidem	Medicamentos	1
Associação de alprazolam, amitriptilina, bisacodil, cafeína, clordiazepóxido, duloxetina, noretisterona, olanzapina e paracetamol	Medicamentos	1
Associação de amitriptilina, diazepam, fluoxetina, haloperidol e prometazina	Medicamentos	1
Associação de bupropiona, carbonato de lítio, clonazepam, mirtazapina, olanzapina e venlafaxina	Medicamentos	1
Associação de carbamazepina e quetiapina	Medicamentos	1
Associação de citalopram, clorpromazina, fluoxetina, nortriptilina e prometazina	Medicamentos	1

## Discussão

Os medicamentos foram os principais agentes implicados entre os casos, seguidos por praguicidas e saneantes domissanitários. Destaca-se a substancial contribuição do emprego simultâneo de substâncias de diferentes grupos de agentes tóxicos nesses casos. A maior parte envolveu o sexo feminino e pessoas com idade entre 15 e 49 anos. As profissões/ocupações mais frequentes foram as seguintes: estudante, desempregado(a) e dona de casa. Os casos ocorreram majoritariamente em Fortaleza, na zona urbana e no ambiente residencial habitual. A via oral foi a mais utilizada. Na maioria dos casos, houve o desenvolvimento de sinais e sintomas clínicos, sem necessidade de internação hospitalar, e o desfecho clínico foi a cura. Os saneantes domissanitários apresentaram a maior taxa de letalidade, seguidos por praguicidas e medicamentos. Os óbitos foram causados por agrotóxicos (inseticidas anticolinesterásicos e herbicidas 2,4-diclorofenoxiacético e glifosato), saneante domissanitário (ácido clorídrico) e medicamentos (ácido valproico e combinação de vários medicamentos psicoativos).

O estudo apresenta limitações, como a análise transversal baseada em dados secundários e o elevado número de campos ignorados ou não preenchidos, revelando falhas no registro. Ademais, os dados foram coletados de uma única unidade de saúde no Ceará, refletindo apenas a realidade de indivíduos que receberam assistência após exposição aos agentes tóxicos. Destaca-se ainda a possível subnotificação de casos de tentativa de suicídio, uma vez que nem todos são atendidos por uma unidade de saúde. Por fim, a ausência de dados clínicos e psicossociais dos pacientes limita a compreensão aprofundada do contexto dessas ocorrências.

Observou-se que o grupo de agentes tóxicos mais implicado nas tentativas de suicídio foi o dos medicamentos. Esse achado concorda com os resultados obtidos por estudos sobre perfil epidemiológico de tentativas de suicídio por intoxicação realizados no Piauí, em 2020 (15), e no Espírito Santo, em 2019 (16).

A alta predominância dos medicamentos ocorre porque provavelmente são, em geral, de livre acesso, baixo custo e aceitos socialmente (2). Além disso, 

a publicidade maciça da indústria farmacêutica e o hábito de estocá-los no domicílio tornam os medicamentos um dos meios mais disponíveis para o emprego nas tentativas de autoextermínio (17).

As principais classes de medicamentos identificadas foram aquelas usadas para o tratamento de transtornos psiquiátricos, seja por contextos de tentativa de autoextermínio por intoxicação medicamentosa (17-19) ou por intoxicação desenvolvida (17). Isso possivelmente acontece porque a maioria dos casos de comportamento suicida estão relacionados a transtornos mentais e sofrimento psíquico, cuja estratégia envolve também a terapia medicamentosa com psicofármacos (18).

Assim, o controle da venda de medicamentos, o descarte correto daqueles fora de uso ou com prazo de validade vencido, o desestímulo à estocagem e o acompanhamento frequente dos pacientes em uso de psicotrópicos pela equipe de saúde são estratégias úteis para reduzir o emprego dos medicamentos nesses casos (20).

O segundo grupo de agentes tóxicos mais frequente entre os registros foi o dos praguicidas. O Brasil, um dos maiores produtores agrícolas do mundo, lidera o consumo de agrotóxicos desde 2008. Ademais, de 2010 a 2020, houve um aumento de 78,3% na quantidade de agrotóxicos comercializados no país (21). 

A disponibilidade desses produtos no meio rural facilita a sua escolha como um meio de autoextermínio. Devido ao expressivo consumo nacional, a aquisição legal de praguicidas é facilitada, tornando-os mais disponíveis. O conhecimento da população sobre a alta letalidade dessas substâncias também incentiva seu uso nas violências autoprovocadas (22).

Nesse cenário, é essencial adotar medidas que restrinjam o acesso aos praguicidas. Por exemplo, nos anos 1990, o Sri Lanka teve uma das maiores taxas de suicídio no mundo. A eliminação gradual de praguicidas no país reduziu essa taxa em cerca de 70% ao longo de 20 anos, salvando cerca de 93 mil vidas (8). 

Os saneantes domissanitários foram o quarto grupo de agentes tóxicos mais implicado entre os registrados. Em um estudo epidemiológico sobre tentativas de suicídio por emprego de agentes tóxicos realizado no mesmo centro, com dados de 2017 e 2018, os saneantes também foram o quarto grupo mais frequente (23). A grande variedade desses produtos em supermercados e a sua presença nas residências e na vida cotidiana da população aumentam o risco de intoxicação e o emprego nas tentativas de suicídio (5).

O sexo feminino foi relatado com maior frequência nos casos, achado que está em conformidade com os resultados de estudos de tentativas de suicídio devido ao uso de agentes tóxicos no Piauí (15), no Espírito Santo (16) e no Ceará (23). O comportamento suicida entre mulheres pode ser atribuído a diversos fatores, como sobrecarga de responsabilidades familiares, exposição à violência doméstica, desigualdade de gênero e às pressões impostas pelo patriarcado (23).

Os agentes tóxicos mais utilizados pelas mulheres foram os medicamentos, provavelmente pela tendência de escolha de métodos menos agressivos, como substâncias tóxicas. Por outro lado, os homens preferem métodos mais violentos, como enforcamento ou arma de fogo. A escolha do método baseado no gênero reflete sua disponibilidade e a aceitabilidade social (23). Assim, a ingestão de medicamentos por mulheres seria socialmente mais aceita do que para os homens, dos quais são esperados métodos mais agressivos pela sociedade, embora os medicamentos sejam igualmente disponíveis para ambos os sexos (24).

A faixa etária mais acometida foi a de 20 a 39 anos. O maior envolvimento de adultos em casos de tentativa de suicídio também foi observado no Piauí (15) e no Espírito Santos (16). De fato, essa fase da vida pode ser marcada por vulnerabilidades, como dificuldades financeiras, problemas no trabalho, conflitos sociais e familiares, abuso de substâncias ilícitas e transtornos psiquiátricos, fatores que influenciam no comportamento suicida (25).

Um número considerável de adolescentes (faixa etária de 15 a 19 anos) também foi identificado. Isso provavelmente ocorre em virtude de esse momento da vida ser marcado por instabilidades biopsicossociais, como cobranças familiares, mudanças acadêmicas e insegurança quanto ao futuro (15,25). 

A ocupação de estudante foi a mais relatada, achado em concordância com os resultados de dois outros estudos epidemiológicos sobre autoextermínio por intoxicação (20,23). Essa situação pode estar relacionada a pressão acadêmica, isolamento social, abuso de substâncias psicoativas e doenças psicológicas que podem caracterizar o período escolar (15). A segunda ocupação mais registrada foi a de pessoas desempregadas. Destaca-se que homens desempregados estão mais predispostos às tentativas de suicídio, possivelmente devido ao aumento do estresse financeiro e à falta de perspectiva profissional (25).

A maior parte dos pacientes envolvidos residia na zona urbana, na região metropolitana de Fortaleza. Isso pode ser explicado pelo fato de o único centro especializado no atendimento a indivíduos expostos a agentes tóxicos do Ceará estar localizado em Fortaleza, o que facilita o acesso ao atendimento para os moradores da capital e arredores, em comparação aos moradores de áreas mais distantes (26).

A exposição aos agentes tóxicos ocorreu principalmente no ambiente domiciliar habitual, ressaltando o papel das relações familiares como fator de risco para o comportamento suicida, podendo existir conflitos parentais, crises conjugais e dificuldades financeiras (26).

Embora, na maioria dos casos de exposição a agentes tóxicos, tenham ocorrido manifestações clínicas, os resultados obtidos mostraram desfecho clínico favorável, com predominância de cura para todos os grupos de agentes tóxicos. O potencial de toxicidade aguda do agente tóxico pode estar relacionado à ocorrência de manifestações clínicas, à necessidade de internação hospitalar da vítima e ao desfecho. Assim, os praguicidas, conhecidos pela expressiva toxicidade aguda, apresentaram maior índice de internação em comparação aos demais grupos. Deve-se considerar que a gravidade do quadro clínico envolve, também, o uso de múltiplas substâncias, a quantidade ingerida, a idade, a história pregressa e o tempo decorrido entre a exposição e o atendimento (26).

Os saneantes domissanitários apresentaram o maior índice de letalidade, e o ácido clorídrico, princípio ativo do ácido muriático, foi o responsável pelos óbitos ocorridos. Esse ácido forte causa destruição tissular por necrose de coagulação e formação de radicais livres, e a extensão do dano depende da concentração da formulação, da duração do contato, da quantidade ingerida e da presença ou ausência de alimentos no estômago (27).

Os inseticidas anticolinesterásicos, como os organofosforados e carbamatos, são responsáveis ​​pelo maior número de óbitos por agrotóxicos (praguicidas de uso agrícola) (12). Eles inibem a acetilcolinesterase, uma enzima responsável por degradar a acetilcolina, o que causa uma hiperestimulação dos receptores colinérgicos, gerando a síndrome colinérgica. Essa síndrome inclui sudorese excessiva, salivação, broncoconstrição, aumento da motilidade gastrointestinal, tremores, entre outros efeitos. A atropina é o antídoto utilizado no tratamento de intoxicações agudas (11).

No Centro de Informação e Assistência Toxicológica do Ceará, o emprego do anticolinesterásico é identificado baseado nas informações fornecidas pelo paciente ou acompanhante, nos sinais e sintomas manifestados e na determinação da inibição da atividade da enzima butirilcolinesterase no plasma, que serve como um indicador biológico de exposição a esses inseticidas (11).

Óbitos foram também causados pelo herbicida ácido 2,4-diclorofenoxiacético. Quando ingerido em grande quantidade, o paciente comumente apresenta odor fenólico, cefaleia, distúrbios gastrointestinais, hemorragia, alterações neuromusculares, letargia, torpor e coma nos casos mais graves (12). Não há antídoto específico para esse herbicida, que em 2015 foi classificado pela International Agency for Research on Cancer como possivelmente carcinogênico para humanos (11).

O glifosato é um herbicida sistêmico, não seletivo e de baixa toxicidade aguda, usado para controle de plantas anuais e perenes. O produto à base de glifosato mais utilizado é o Roundup, em cuja formulação é empregada a polioxetilenoamina como surfactante. Foi relatado que essa substância pode aumentar a permeabilidade celular ao glifosato (11). 

A intoxicação aguda decorrente da exposição ao glifosato resulta em sintomas gastrointestinais leves, enquanto as intoxicações moderadas ou graves se manifestam com sangramento gastrointestinal, hipotensão, disfunção pulmonar e dano renal. Não há antídoto específico para esse herbicida (12), classificado pela International Agency for Research on Cancer como um provável carcinógeno para humanos em 2015 (11).

Os medicamentos apresentaram o menor índice de letalidade entre os grupos estudados, o que é compatível com o fato de que somente um dos casos de óbito ter sido causado por um único agente, o ácido valproico. Todos os outros foram devidos à ingestão simultânea de vários medicamentos. O efeito da ação combinada de vários agentes propicia a ocorrência de intoxicações de maior gravidade, inclusive fatais, como observado nos casos avaliados (11).

Cabe ressaltar o papel da atenção primária à saúde na identificação, avaliação, manejo e encaminhamento de casos relacionados ao comportamento suicida, desenvolvendo ações voltadas à identificação precoce e à intervenção oportuna. A sua integração com os serviços de saúde mental fortalece o suporte psicossocial e garante o acompanhamento adequado desses casos. O acesso a tratamentos especializados, associado a redes de apoio à saúde mental, auxilia na redução do sofrimento psíquico e na prevenção de novas tentativas de suicídio (28).

Ao apresentar o perfil dos indivíduos e os agentes tóxicos implicados nas tentativas de suicídio registradas no Centro de Informação e Assistência Toxicológica do Ceará, o presente estudo contribui com conhecimento necessário para nortear políticas e medidas de prevenção dessas ocorrências. Entre elas, destacam-se a regulamentação do acesso aos agentes tóxicos, o fortalecimento de programas de apoio psicológico e a ampliação de campanhas de prevenção ao suicídio. Assim, o estudo colabora para a redução das tentativas de suicídio e para a proteção às populações vulneráveis identificadas.

## Data Availability

O banco de dados e os códigos de análise utilizados na pesquisa estão disponíveis em: https://data.scielo.org/dataset.xhtml?persistentId=doi:10.48331/scielodata.CAI7TX.
